# Lysine methyltransferase G9a is not required for DNMT3A/3B anchoring to methylated nucleosomes and maintenance of DNA methylation in somatic cells

**DOI:** 10.1186/1756-8935-5-3

**Published:** 2012-01-27

**Authors:** Shikhar Sharma, Daniel S Gerke, Han F Han, Shinwu Jeong, Michael R Stallcup, Peter A Jones, Gangning Liang

**Affiliations:** 1 Department of Biochemistry and Molecular Biology, USC/Norris Comprehensive Cancer Center, Keck School of Medicine, University of Southern California, Los Angeles, CA 90089-9181, USA; 2 Department of Urology, USC/Norris Comprehensive Cancer Center, Keck School of Medicine, University of Southern California, Los Angeles, CA 90089-9181, USA; 3 Program in Genetic, Molecular and Cellular Biology, USC/Norris Comprehensive Cancer Center, Keck School of Medicine, University of Southern California, Los Angeles, CA 90089-9181, USA; 4 Department of Pharmacology and Pharmaceutical Sciences, USC/Norris Comprehensive Cancer Center, Keck School of Medicine, University of Southern California, Los Angeles, CA 90089-9181, USA

**Keywords:** G9a, DNMT3A, DNMT3B, DNA methylation, Nucleosome, Maintenance, Epigenetics

## Abstract

**Background:**

DNA methylation, histone modifications and nucleosome occupancy act in concert for regulation of gene expression patterns in mammalian cells. Recently, G9a, a H3K9 methyltransferase, has been shown to play a role in establishment of DNA methylation at embryonic gene targets in ES cells through recruitment of *de novo *DNMT3A/3B enzymes. However, whether G9a plays a similar role in maintenance of DNA methylation in somatic cells is still unclear.

**Results:**

Here we show that G9a is not essential for maintenance of DNA methylation in somatic cells. Knockdown of G9a has no measurable effect on DNA methylation levels at G9a-target loci. DNMT3A/3B remain stably anchored to nucleosomes containing methylated DNA even in the absence of G9a, ensuring faithful propagation of methylated states in cooperation with DNMT1 through somatic divisions. Moreover, G9a also associates with nucleosomes in a DNMT3A/3B and DNA methylation-independent manner. However, G9a knockdown synergizes with pharmacologic inhibition of DNMTs resulting in increased hypomethylation and inhibition of cell proliferation.

**Conclusions:**

Taken together, these data suggest that G9a is not involved in maintenance of DNA methylation in somatic cells but might play a role in re-initiation of *de novo *methylation after treatment with hypomethylating drugs, thus serving as a potential target for combinatorial treatments strategies involving DNMTs inhibitors.

## Background

DNA methylation is an essential epigenetic gene silencing mechanism which interplays with histone modifications and nucleosome occupancy for regulation of tissue-specific gene expression patterns and chromatin architecture in mammalian cells [1]. DNA methylation patterns are established during embryogenesis and then faithfully maintained in differentiated tissues, enabling preservation of cellular identity through multiple somatic divisions. Failure in proper maintenance of methylation patterns can result in development of disease states such as cancer [2,3]. Thus, it is essential to faithfully maintain DNA methylation patterns in differentiated cells through somatic divisions.

In mammals, the *de novo *DNA methytransferases, DNMT3A/3B, primarily establish the methylation patterns during embryonic development [4] and later maintain them in differentiated tissues through cooperation with the maintenenace DNA methyltransferase, DNMT1 [5-7]. Recent studies by our group and others have revealed distinct maintenance mechanisms used by these enzymes for preservation of methylation patterns in somatic cells [8,9]. While DNMT1 transiently interacts with the chromatin and primarily performs its maintenance activity in association with the replication fork, DNMT3A/3B remain preferentially bound to nucleosomes in chromatin regions containing methylated repeats and CpG islands, which stabilizes these proteins and assists in faithful propagation of DNA methylation within the methylated domains through cooperative activity of DNMT3A/3B and DNMT1 enzymes [9-11]. However, the mechanisms responsible for preferential targeting of DNMT3A/3B to such methylated regions are still poorly understood.

In embryonic stem (ES) cells, targeting of *de novo *DNMT3A/3B enzymes to specific chromatin regions involves interactions with auxiliary factors in addition to their direct interactions with the nucleosomes [12,13]. DNMT3A/3B interact with various chromatin-associated proteins including heterochromatin protein 1 (HP1), histone deacetylase 1 (HDAC1), UHRF1 and histone methyltransferases such as EZH2, suggested to play a role in recruitment of DNMT3A/3B to specific chromatin regions for *de novo *methylation in ES cells [9,13,14]. However, recent studies suggest that these auxiliary proteins are not required for interaction of DNMT3A/3B with the nucleosomes in somatic cells suggesting existence of other mechanisms involved in association of DNMT3A/3B with chromatin [10].

H3K9 methylation has also been established to interplay with DNA methylation for gene silencing in cells. In *neurospora crassa*, H3K9 methylation directs DNA methylation to transposable elements [15,16]. A similar link between H3K9 methylation and DNA methylation is present in mammals where heterochromatic H3K9 methyltransferase, Suv39h1, has been shown to direct DNA methylation to major satellite repeats at pericentric heterochromatin [17]. Recently, G9a, a euchromatic H3K9 methyltransferase, has also been found to direct DNA methylation to H3K9 methylated regions in ES cells [18]. G9a, along with its partner protein GLP, is crucial for H3K9 (mainly H3K9me and H3K9me2) methylation of euchromatin and is involved in transcriptional silencing [19,20]. G9a binds to its own product, H3K9me and H3K9me2 residues, through its ankyrin domain, a mechanism suggested to play a role in propagation of H3K9 methylation through cell divisions [21,22]. Recent studies have shown that G9a physically interacts with Dnmt3a/3b and recruits them to G9a-target gene promoters, retrotransposons and major satellite repeats for *de novo *methylation in ES cells [23], independent of its histone methyltransferase activity [24]. While an essential role of G9a in directing *de novo *DNA methylation in ES cells has been established, whether it plays a similar role in maintenance of DNA methylation patterns in somatic cells remains unclear.

Here we show that G9a is not required for anchoring of DNMT3A/3B to nucleosomes in methylated chromatin regions and for maintenance of DNA methylation in somatic cells. Using sucrose gradient chromatin fractionation analysis, we show that G9a strongly binds to both mononucleosomes and polynucleosomes, similar to DNMT3A/3B. However, knockdown of G9a in somatic cells does not decrease binding of DNMT3A/3B to nucleosomes and no discernible reduction in DNA methylation levels of G9a-associated target genomic regions occurs even in the absence of G9a, suggesting no role of G9a in maintenance of DNA methylation. However, G9a-knockdown renders cancer cells more sensitive to 5-Aza-CdR (5-aza-2'-deoxycytidine) treatment resulting in increased DNA hypomethylation and cell growth inhibition, indicating that G9a might be involved in re-initiation of *de novo *methylation and thus could serve as a promising target for combinatorial cancer treatment strategies involving DNA hypomethylating drugs.

## Results

### G9a strongly associates with polynucleosomes similar to DNMT3A/3B

**Figure 1:**
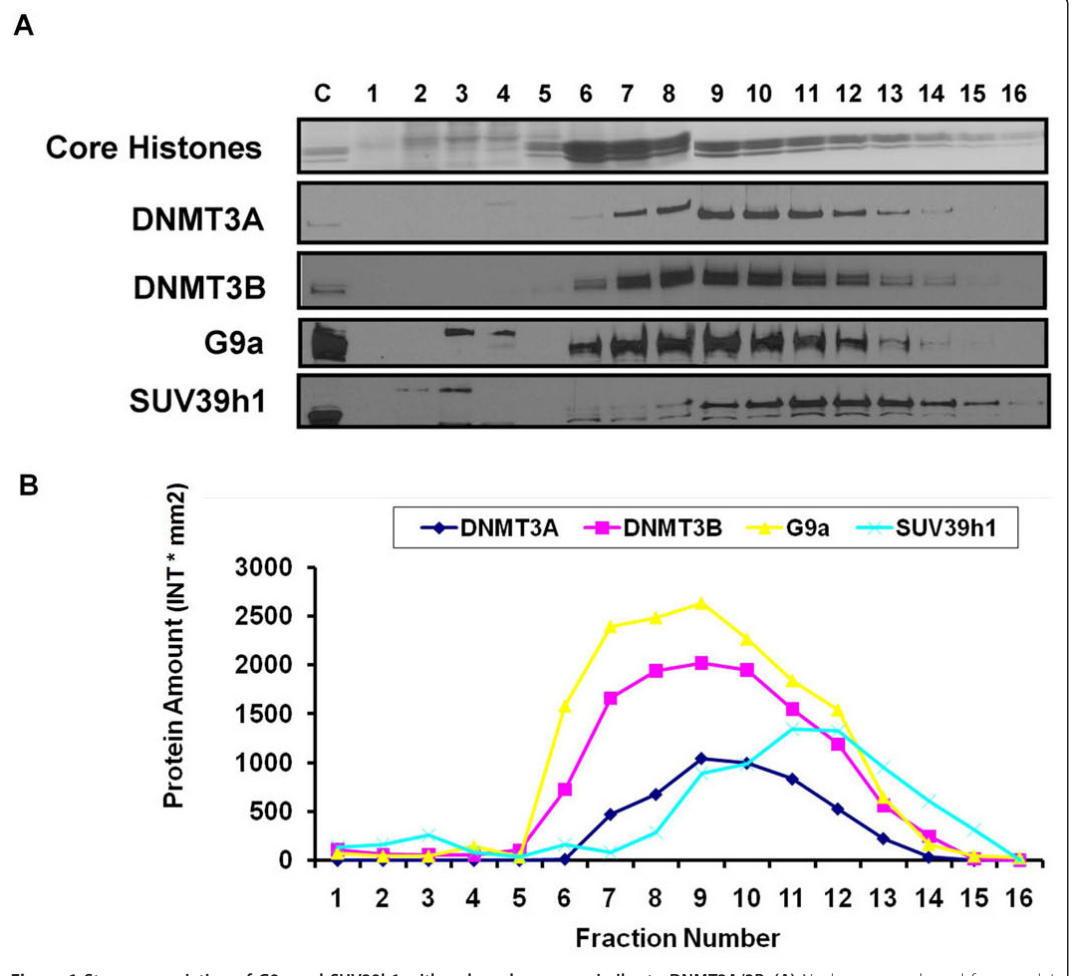
**Strong association of G9a and SUV39h1 with polynucleosomes similar to DNMT3A/3B**. **(A) **Nucleosomes released from nuclei partially digested with MNase at low ionic strength were resolved by ultracentrifugation on a sucrose density gradient (5% to 25%) containing 300 mM NaCl. Gradients were fractionated and analyzed as described in the Methods section. Ponceau S staining shows core histones transferred onto the membrane from the SDS/PAGE gel. The control lanes (denoted as C) on the gels were loaded with unfractionated nuclear extract loaded on the gels to monitor the quality of the immunostaining of the membranes. **(B) **Quantitation of protein bands obtained from the western blot was done using Quantity One software (Bio-Rad). Plotting of levels of individual proteins in each fraction shows co-sedimentation of G9a and DNMT3A/3B while SUV39h1 shows a sedimentation profile shifted towards bottom of the gradient indicating association with heavier condensed heterochromatin fragments.

To test the role of G9a, the euchromatic histone methyltransferase, in the strong nucleosome anchoring manifested by DNMT3A/3B, we examined their nucleosomal binding patterns using sucrose density gradient analysis. We subjected purified HCT116 nuclei to partial digestion with MNase, which cuts linker DNA to generate nucleosomal fragments of various sizes, yielding a mixture of mono- and poly-nucleosomes. The nucleosomal digests were then fractionated on sucrose gradients containing 300 mM NaCl and the distribution of chromatin-associated proteins analyzed through immunoblotting (Figure 1A). G9a associated strongly with polynucleosomes with substantial amounts of G9a protein sedimenting in nucleosome-containing fractions, possibly in association with its own enzymatic products, H3K9me and H3K9me2 [21]. Strikingly, the sedimentation profile of G9a was very similar to that of DNMT3A/3B (Figure 1B). Interestingly, SUV39h1, the heterochromatic histone methyltransferase, was also found associated with the polynucleosomes. However, its sedimentation profile was slightly shifted towards the bottom fractions of the gradient, containing the larger chromatin fragments indicating association with the condensed heterochromatin [17,25,26] (Figure 1A, B). Strong association of DNMT3A/3B and histone methyltransferases with nucleosomes is in agreement with the inheritance model recently proposed for DNA methylation and histone modifications where the enzymes remain associated with their products to enable their proper propagation [8,21,26,27].

### G9a tightly binds to intact mononucleosomes

**Figure 2:**
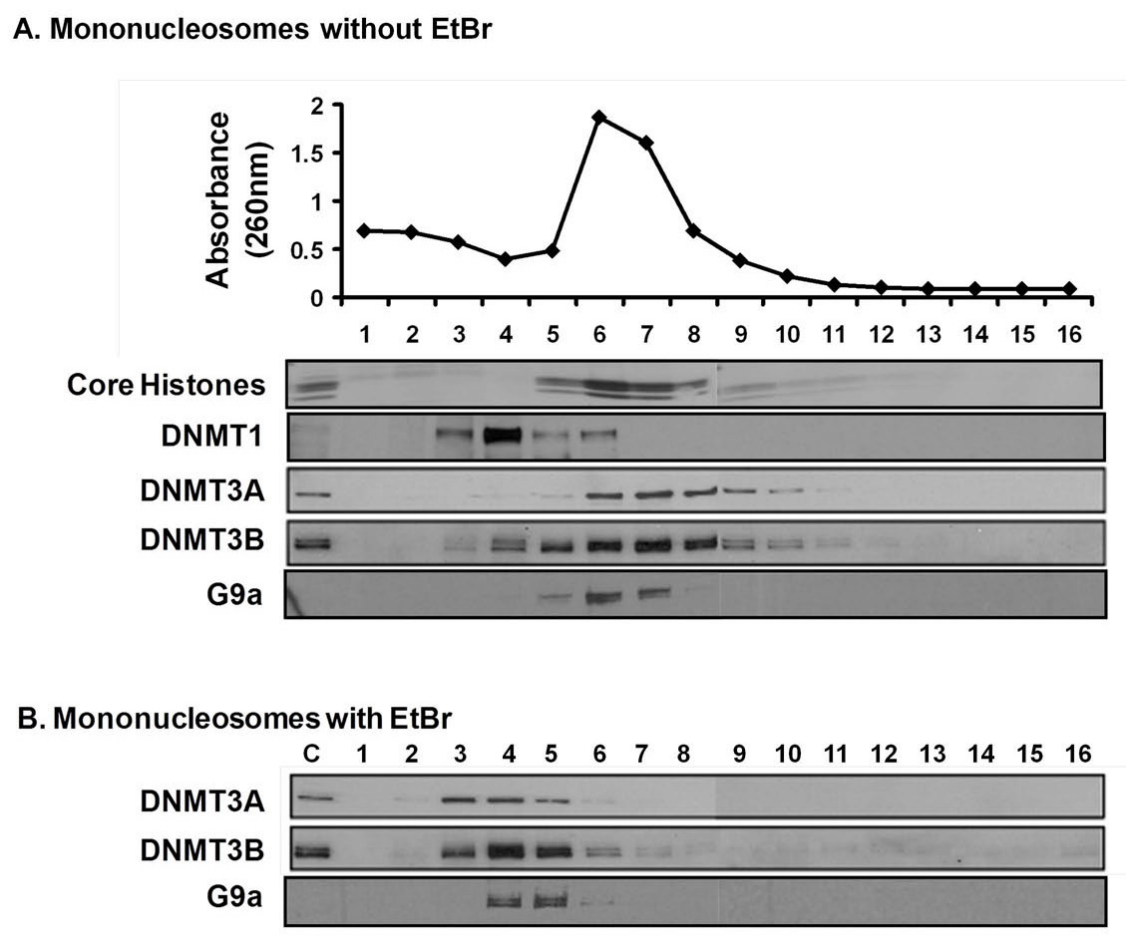
**G9a along with DNMT3A/3B binds to intact nucleosomal structures**. Mononucleosomes released by extensive digestion with MNase were incubated in the absence **(A) **or presence **(B) **of 300 μg/ml EtBr for 10 min at room temperature, before loading onto the sucrose density gradients (5% to 25%) containing 300 mM NaCl. In **(A)**, absorbance of each fraction, read at 260 nm, has been plotted on the top to represent the amount of DNA sedimenting in each fraction. Gradients were fractionated and analyzed as described previously.

We next asked whether G9a could bind to mononucleosomes in a manner similar to that previously observed for DNMT3A/3B. Mononucleosomal MNase digests from HCT116 cells were analyzed on sucrose gradients containing 300 mM NaCl. Mononucleosomes containing approximately 146 bp DNA fragments and core histones localized in a peak at fraction 6 (Figure 2A). DNMT1 dissociated from nucleosomes, forming a peak at fraction 4, while DNMT3A/3B formed a peak at fraction 7 suggesting that DNMT3A/3B are bound to mononucleosomes and that their presence altered the sedimentation of bound nucleosomes by one fraction relative to bulk mononucleosomes. G9a also remained tightly anchored to the mononucleosomes similar to DNMT3A/3B enzymes. SUV39h1 also displayed strong binding to mononucleosomes (Additional file 1, Figure S1). However, under such extensive digestion of chromatin, a substantial portion of cellular SUV39h1 protein dissociated from the nucleosomes, possibly due to disruption of condensed heterochromatin structure [25]. The sedimentation profiles of G9a and SUV39h1 showed marked changes when the extent of MNase digestion was altered from partial to extensive (Figure 1, 2A, S1), similar to that observed previously for DNMT3A/3B [10], indicating physical association of these proteins with nucleosomes. Similar binding of G9a and SUV39h1 to mononucleosomes was also observed in 293T cells indicating that the strong nucleosomal association of these proteins takes place in both cell types and is not due to potential cell type-specific interactions (Additional file 1, Figure S1).

To further explore whether these enzymes require intact nucleosomal structures for their association with chromatin, we performed the sucrose gradient analysis on the mononucleosomes treated with ethidium bromide (EtBr) which disrupts the nucleosomal structure by intercalating into DNA but does not interfere with the protein-protein interactions [17,28-30]. Mononucleosomes were incubated with EtBr prior to loading onto sucrose gradients containing 300 mM NaCl (Figure 2B). The distribution of G9a changed dramatically, similar to DNMT3A/3B, upon disruption of nucleosomal structure by EtBr with the enzymes now sedimenting mainly in factions 3 to 5, which do not contain measureable histone components of the nucleosome. These data show that G9a and DNMT3A/3B enzymes require intact nucleosomal structures for their association with chromatin.

### G9a is not essential for maintenance of DNA methylation in somatic cells

**Figure 3:**
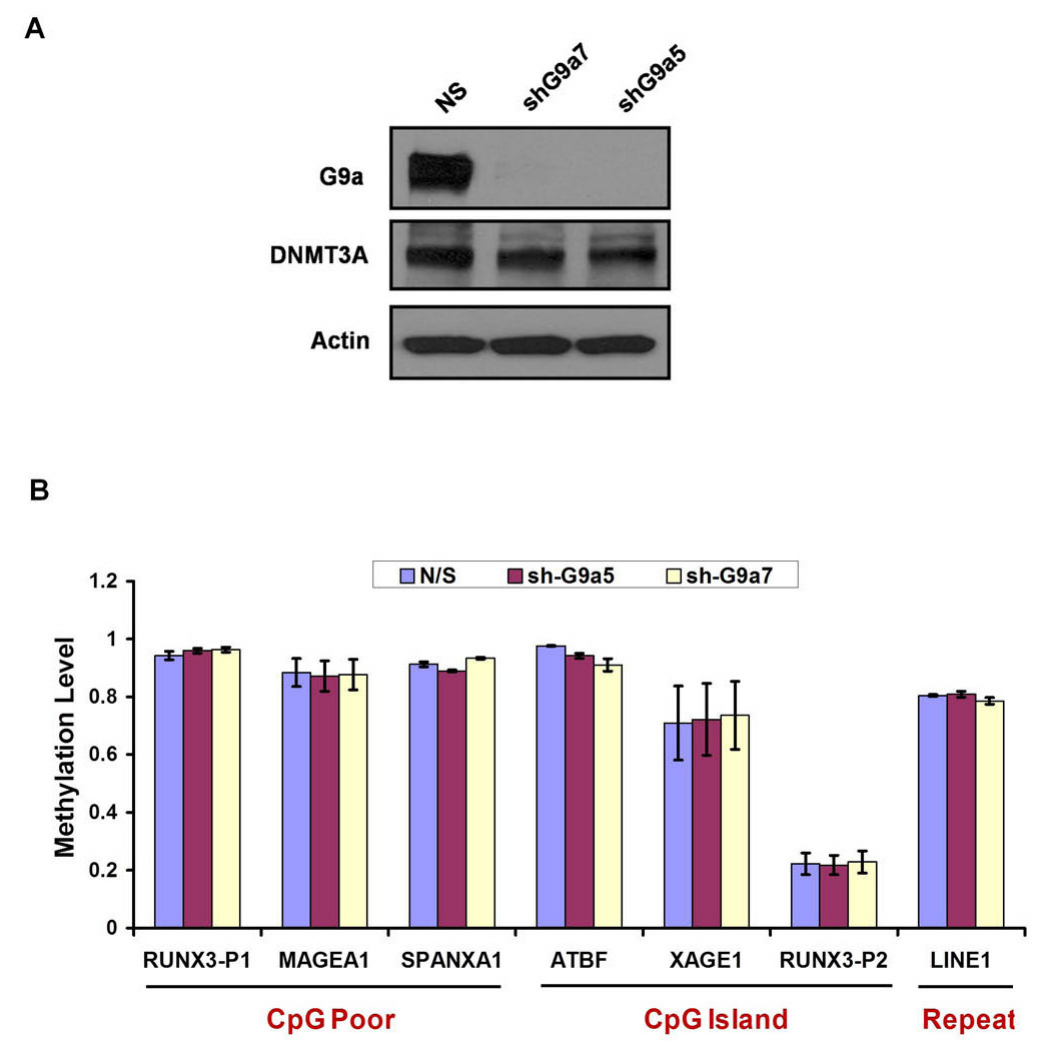
**Depletion of G9a does not impair maintenance of DNA methylation in somatic cells**. **(A) **Western blot of nuclear extracts from HCT116 cells infected with either control (NS) shRNA or shRNAs against G9a (shG9a5 or shG9a7), prepared 14 days after infection. **(B) **The levels of DNA methylation at different loci in HCT116 cells infected with either control (NS) shRNA or shRNAs against G9a (shG9a5 or shG9a7) were measured through Ms-SNuPE 14 days after infection. Data represent mean and SEM of methylation levels measured at three independent CpG sites within each locus.

Recently, G9a was shown to direct DNA methylation to retrotransposons, major satellite repeats and densely methylated CpG-rich promoters in ES cells through recruitment of DNMT3A/3B proteins [23]. To ascertain whether a similar role of G9a exists in maintenance of DNA methylation in somatic cells, we knocked down G9a in HCT116 cells using shRNA constructs. G9a protein levels were severely reduced in G9a shRNA (shG9a5, shG9a7) infected cells compared to the non-specific shRNA (NS) control infected cells (Figure 3A). DNMT3A protein levels did not show any difference among the infected cell lines. Next we examined DNA methylation levels at various CpG poor and CpG island promoter regions and repeats in G9a knockdown (G9a-kd) and control infected HCT116 cells. We selected six highly methylated regions including some previously verified G9a-target regions in HCT116 cells [31] (CpG poor promoters: *RUNX3P1, MAGE-A1, SPANXA1*; CpG island promoters: *ATBF1, XAGE1*; Repeats: *LINE1*) and one unmethylated region (CpG island: *RUNX3P2*) for our analysis [18-20,23,32]. Cooperative activity of DNMT3A/3B and DNMT1 has been previously shown to be required for maintenance of DNA methylation at these methylated loci in HCT116 cells [33-35]. We did not observe any substantial change in DNA methylation levels at the analyzed regions in the G9a-kd cells compared to control infected HCT116 cells (Figure 3B). These data suggest that unlike in ES cells, G9a is not essential for maintenance of DNA methylation at these loci in somatic cells, in agreement with some other recent studies [31,36].

### DNMT3A/3B do not require G9a for anchoring to nucleosomes

**Figure 4:**
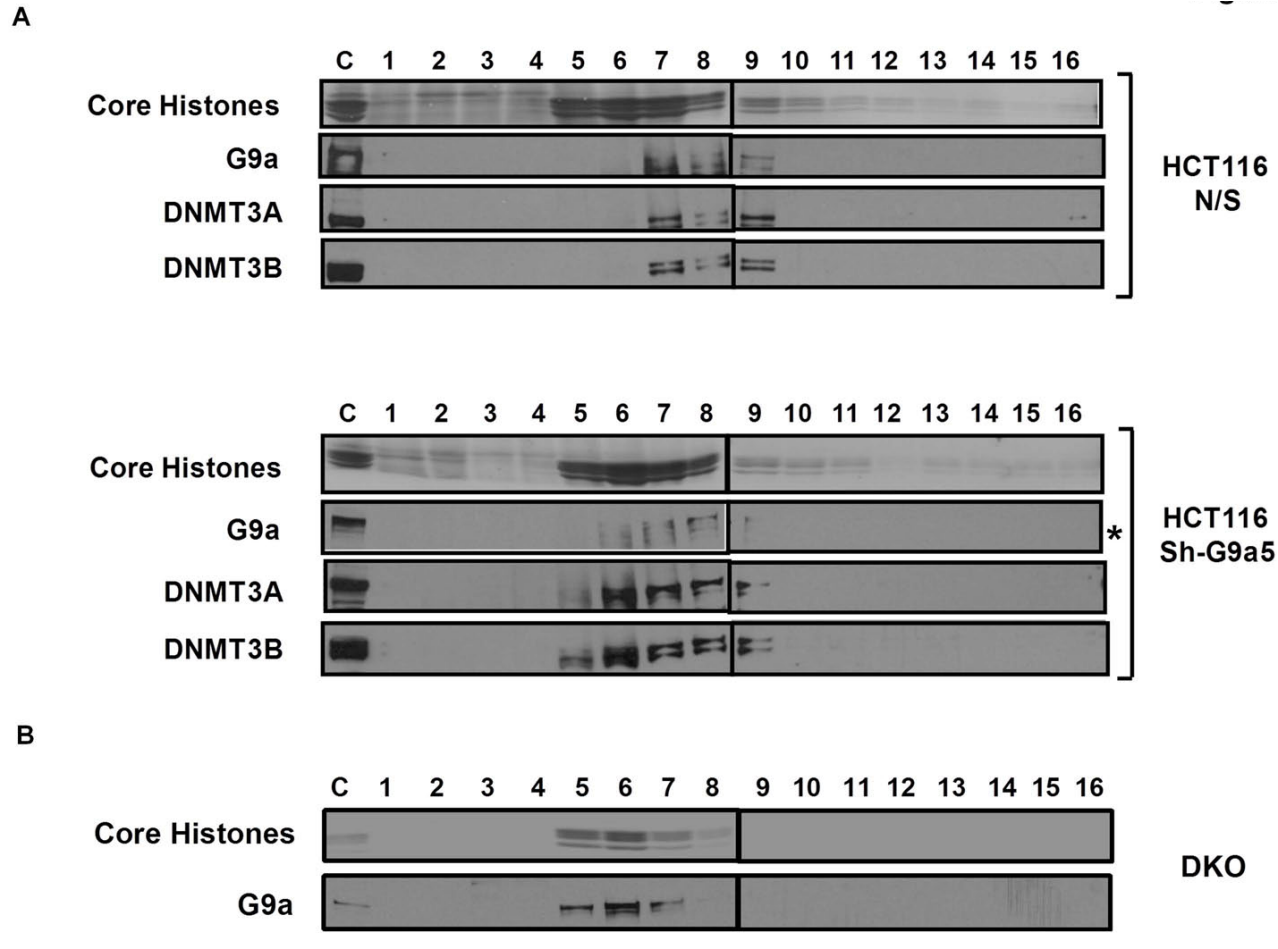
**DNMT3A/3B and G9a do not require each other for anchoring to nucleosomes in somatic cells**. Mononucleosomal digests prepared by extensive MNase digestion of nuclei from **(A) **HCT116 cells infected with either control NS shRNA or shG9a5 shRNA as shown in Figure 3 and **(B) **hypomethylated DKO1 cells, were resolved by ultracentrifugation on a sucrose density gradient (5% to 25%) containing 300 mM NaCl. Gradients were fractionated and analyzed as described previously. Western blot was highly overexposed for detecting residual G9a protein in the shG9a5 shRNA infected HCT116 cells. * Overexposed.

The finding that DNA methylation is maintained in the absence of G9a in somatic cells prompted us to examine whether DNMT3A/3B, which are recruited and anchored to target chromatin regions by G9a for DNA methylation in ES cells [23,24], can still strongly associate with nucleosomes even in the absence of G9a in somatic cells. Since DNMT3A/3B cooperate with DNMT1 for maintenance of DNA methylation at various methylated genomic loci in somatic HCT116 cells, including the G9a-target loci examined in this study [33], their continued association with such loci might enable faithful propagation of DNA methylation even in the absence of G9a. We analyzed mononucleosomal digests from HCT116 cells infected with either G9a shRNA or non-specific control on sucrose gradients containing 300 mM NaCl. DNMT3A/3B along with G9a were tightly associated with nucleosomes in the control cells as expected (Figure 4A). Interestingly, even in G9a knockdown cells, DNMT3A/3B remained strongly bound to the nucleosomes indicating their continued association with the target loci even in the absence of G9a. Taken together, these data indicate that in somatic cells, the strong association of DNMT3A/3B with methylated chromatin regions is not mediated by G9a and DNMT3A/3B, in cooperation with DNMT1, can faithfully maintain DNA methylation at their target loci independent of G9a. We also asked whether impaired DNA methylation could feed back on H3K9 methylation, as previously suggested [37], and disrupt binding of G9a to H3K9 methylated regions. Mononucleosomal digests from severely hypomethylated DNMT-deficient DKO1 (*DNMT1*^ΔE2-5^, *DNMT3B *^-/- ^) cells, containing severely reduced levels of DNMT3A [11,33], were analyzed on sucrose gradients containing 300 mM NaCl. The majority of G9a was found tightly anchored to nucleosomes even in DKO1 cells indicating that a loss of DNA methylation and absence of DNMTs does not affect G9a's association with the chromatin which is in agreement with a previous study [18] (Figure 4B). Interestingly, we did observe a slight shift towards the top of the gradient in sedimentation profiles of DNMT3A/3B in G9a-kd cells and G9a in DNMT-deficient DKO1 cells, with the proteins sedimenting a fraction higher on the gradient which might result from the absence of respective chromatin-associated proteins in these cells. We have previously shown that binding of chromatin-associated enzymes like DNMT3A/3B with mononucleosomes alters the sedimentation of bound nucleosomes by one fraction relative to bulk mononucleosomes towards the bottom of the gradient (DNMT3A/3B form a peak in fraction 7 compared to peak of core histones in fraction 6 (Figure 2A)) due to an increase in molecular weight of the enzymes-bound nucleosomes [10]. Thus while in HCT116 N/S cells, mononucleosomes bound to G9a and DNMT3A/3B (possibly bound as part of a common protein complex) form a peak in fraction 7 of the gradient, absence of either of these nucleosome-binding proteins in HCT116 G9a-kd cells and DNMT-deficient DKO1 cells results in a decrease in molecular weight of such enzyme-bound nucleosomes and hence a shift in their sedimentation profile towards the top of the gradient forming a peak in fraction 6. However, our gradient data clearly suggests that while G9a and DNMT3A/3B might bind to nucleosomes in similar chromatin regions, their nucleosome binding is independent of presence of each other.

**Figure 5:**
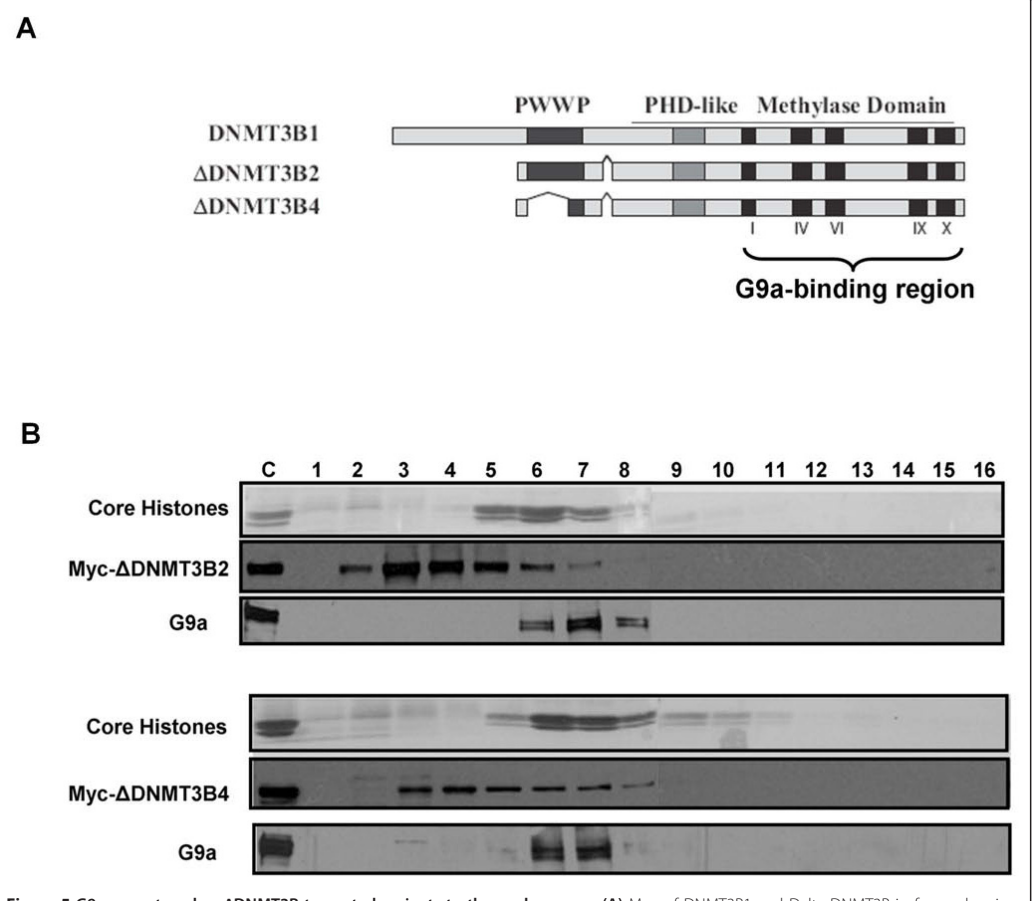
**G9a cannot anchor ΔDNMT3B truncated variants to the nucleosomes**. **(A) **Map of DNMT3B1 and Delta DNMT3B isoforms showing the PWWP and PHD-like domains located in the N-terminal regions, and the catalytic methylase domains in the C-terminal region. **(B) **Mononucleosomal digests prepared by extensive MNase digestion of nuclei from 293T cells expressing either ΔDNMT3B2 or ΔDNMT3B4, were resolved by ultracentrifugation on a sucrose density gradient (5% to 25%) containing 300 mM NaCl. Gradients were fractionated and analyzed through immunoblotting as described previously.

Next, we asked whether G9a could target the truncated ΔDNMT3B variants, which lack parts of N-terminal domain of DNMT3B including the PWWP domain but possess a conserved active catalytic domain, to nucleosomes (Figure 5A). The truncated ΔDNMT3B variants have been associated with aberrant promoter methylation in non-small cell lung cancer (NSCLC) cell lines, possibly arising from their aberrant targeting due to a reduction in their chromatin binding affinities [10,38,39]. G9a associates with DNMT3A/3B through the interaction of its ankyrin domain with the catalytic domains of DNMT3A/3B [23], therefore it should be able to physically interact and recruit catalytically active ΔDNMT3B variants to the target chromatin regions. Mononucleosomal digests from 293T cells expressing Myc-tagged ΔDNMT3B variants were analyzed on sucrose gradients containing 300 mM salt. While G9a remained tightly bound to the nucleosomes, the delta DNMT3B variants completely dissociated from the nucleosomes (Figure 5B). Taken together, these data suggest that unlike in ES cells, where G9a is essential for DNA methylation through recruitment of DNMT3A/3B enzymes to the H3K9 methylated chromatin regions, G9a is not essential for anchoring of DNMT3A/3B to the nucleosomes and for propagation of DNA methylation in somatic cells.

### G9a knockdown synergizes with 5-Aza-CdR treatment resulting in increased DNA hypomethylation and inhibition of cell growth

**Figure 6:**
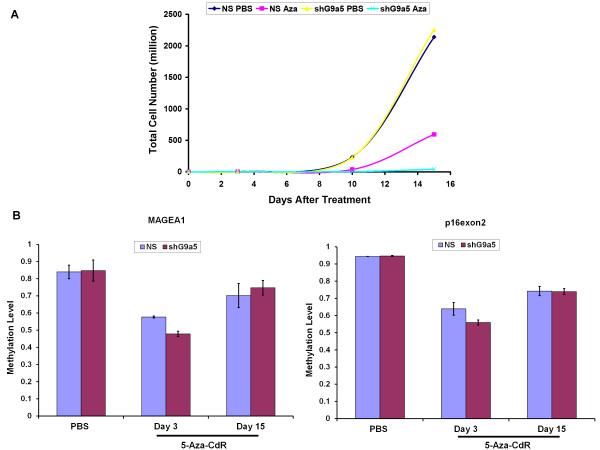
**Increased DNA hypomethylation and growth inhibition by 5-Aza-CdR in G9a knockdown cells**. **(A) **Cell growth curve and **(B) **DNA methylation levels at *MAGE-A1 *and *p16exon2 *locus, of G9a-knockdown (shG9a5) and control (NS) HCT116 cells treated with either PBS or 5-Aza-CdR. Cells were treated with the drug for 24 h. The drug containing media was then replaced with fresh media. The cells were kept in culture and cell counts and DNA methylation levels were assessed at respective time points. For 5-Aza-CdR treatment, samples examined were taken after 3 (Day 3) and 15 (Day 15) days of drug treatment. For PBS control treatment, sample examined was taken after 3 days of drug treatment. DNA methylation levels were measured using Ms-SNuPE assay as described in the Methods section. Data represent mean and range of methylation levels from two independent biological replicate drug treatment experiments, in each measuring methylation levels at three independent CpG sites within each locus.

While our results suggested no role of G9a in maintaining DNA methylation in somatic cells, it remained plausible that G9a might be involved in re-initiation of *de novo *DNA methylation (rebound methylation) at hypomethylated sites after treatment with hypomethylating drugs, a phenomenon commonly observed after treatment of cancer cells with DNA methylation inhibitors [33]. Since G9a plays a role in directing *de novo *DNA methylation to various G9a target regions in ES cells [23], a similar role of G9a in directing rebound methylation in somatic cells is possible. Therefore, we hypothesized that knockdown of G9a might synergize with treatment with DNMTs inhibitors enabling greater reduction in DNA methylation through inhibition of interplay between the histone methylation and DNA methylation machineries. To test this hypothesis, we treated G9a shRNA (shG9a5) and non-specific control (NS) infected HCT116 cells with 5-Aza-CdR (5-aza-2'-deoxycytidine). Strikingly, G9a-kd cells treated with 5-Aza-CdR showed substantially greater inhibition of cell proliferation compared to 5-Aza-CdR treated control infected HCT116 cells (Figure 6A). Analysis of DNA methylation at the *MAGE-A1 *promoter, a G9a-target locus, and *p16exon2*, 72 hrs after drug treatment showed greater reduction in DNA methylation at both loci in G9a-kd cells compared to control infected cells (Figure 6B). The increased hypomethylation of *MAGE-A1 *locus in 5-Aza-CdR treated G9a-kd cells compared to non-specific control treated cells was further confirmed by bisulfite sequencing showing a decrease in *MAGE-A1 *promoter methylation to 45% in G9a-kd cells compared to 55% in NS control cells after drug treatment (Additional file 2, Figure S2). Since both G9a-kd and control HCT116 cells displayed similar growth rates in mock PBS treatments, the increased hypomethylation caused by 5-Aza-CdR in G9a-kd cells compared to control cells was not due to differential incorporation of the drug into replicating DNA in these cells (Figure 6A). However, while we did see reduced methylation levels 72 hrs after 5-Aza-CdR treatment, DNA methylation levels recovered to their initial high levels in both G9a-kd and control HCT116 cells with increased time in culture (Day 15), possibly due to the activity of residual DNMT3A/3B enzymes still associated with such regions and the gradual recovery in levels of cellular DNMT3A/3B and DNMT1 enzymes in absence of the hypomethylating drug [11,40]. After observing the pronounced inhibition of cell proliferation by 5-Aza-CdR upon depletion of G9a, we also tried pharmacologic inhibition of G9a by performing a double treatment using a G9a-inhibitor, BIX-01294 [41] in combination with 5-Aza-CdR. However, BIX-01294 was very toxic to cells, as previously discussed [42], and thus could not be used for combination treatment. Taken together, these data suggest that G9a knockdown synergizes with DNMTs inhibition leading to higher cell growth inhibition and DNA hypomethylation and G9a might serve as an effective target for combinatorial cancer treatment strategies involving DNMTs inhibitors.

## Discussion

We have previously shown that the majority of DNMT3A/3B strongly anchor to nucleosomes containing methylated DNA in somatic cells. Such binding, which requires the presence of DNA methylation, further stabilizes these proteins [10,11]. Based on these data, we proposed a revised inheritance model for DNA methylation where DNMT3A/3B remain associated with methylated chromatin domains i.e. in association with their product 5-methylcytosine enabling proper maintenance of DNA methylated states through somatic divisions in cooperation with DNMT1 [8]. This model is similar to proposed inheritance models for histone marks where the histone methyltransferases remain associated with the chromatin domains containing their own mark enabling faithful maintenance [21,26,27]. The strong binding of DNMT3A/3B and H3K9 methyltransferases, G9a and SUV39h1, to nucleosomes observed in our sucrose density gradient experiments strongly supports the existence of such proposed inheritance models. Whether a common link exists between these two similar inheritance models for H3K9 methylation and DNA methylation is still unclear. H3K9 methylation and DNA methylation patterns are found to be highly coincident in mammalian cells [14,43,44] suggesting the possibility of interplay between these two epigenetic machineries. Indeed recent studies revealed such a link between these two mechanisms in ES cells where G9a was shown to direct DNA methylation to certain loci in ES cells [9]. However, the presence of a similar role of G9a in directing maintenance of DNA methylation in somatic cells is still inconclusive.

Here we have shown that unlike in ES cells, G9a is not essential for propagation of DNA methylation in somatic cells since depletion of G9a in somatic cells did not impair maintenance of DNA methylation at G9a-target loci. Our data are in agreement with some other recent studies indicating that while G9a is essential for *de novo *methylation, it is dispensable for maintenance of DNA methylation [31,36]. In addition, our data provide insights into possible mechanisms responsible for such G9a-independent maintenance of DNA methylation in somatic cells. We have shown that DNMT3A/3B remain bound to methylated chromatin regions even in the absence of G9a indicating that these enzymes do not require G9a for their presence at silent methylated domains and they can faithfully maintain DNA methylation in somatic cells independent of G9a.

Based on previous studies in ES cells and our current data, we propose that G9a primarily plays a role in establishment of DNA methylation at target loci in ES cells by recruiting DNMT3A/3B for *de novo *methylation at such regions. G9a may act in concert with DNMT3L, a regulatory factor expressed only in ES cells which stimulates DNMT3A/3B activities [45], in the *de novo *methylation process. However, once the methylation patterns have been established, DNMT3A/3B remain stably associated with nucleosomes in methylated chromatin regions in somatic cells, independent of G9a, and faithfully propagate DNA methylation in such domains in cooperation with DNMT1 through multiple somatic divisions. One possible reason for such differential effects of G9a on DNA methylation observed in ES cells compared to somatic cells might be the greater level of 'epigenetic plasticity' in the ES cells. ES cells require a plastic epigenome since they need to reprogram themselves along specific lineages [46]. In contrast, differentiated somatic cells possess a more restricted chromatin structure which they need to faithfully maintain to preserve their cellular identity through somatic divisions [46]. Therefore, in somatic cells, DNMT3A/3B enzymes remain stably associated with the previously methylated chromatin domains ensuring faithful maintenance of methylated states without causing any aberrant *de novo *methylation [11]. Interestingly, we also found that the presence/absence of DNA methylation does not affect association of G9a, which binds to H3K9me and H3K9me2 residues, with nucleosomes. While DNMT3A/3B require DNA methylation for their binding to nucleosomes [11], our current data suggest that impaired DNA methylation does not affect G9a's association with H3K9 methylated chromatin regions and distinct mechanisms are involved in localization of these enzymes in somatic cells.

Our data also show that while G9a is not required for maintenance of DNA methylation in differentiated cells, simultaneous targeting of G9a and DNMTs results in increased inhibition of cell proliferation and greater DNA hypomethylation. The striking increase in inhibition of cell proliferation observed in our experiments highlights the strong potential of combinatorial cancer treatment strategies targeting repressive histone methylation and DNA methylation machineries together. The pronounced cell growth inhibition observed upon simultaneous knockdown of G9a along with treatment with 5-Aza-CdR might be a result of widespread changes in DNA methylation and accompanying changes in histone modifications [47]. Our data also show that such combinatorial targeting approach further increases the efficacy of the DNA hypomethylating drugs in reducing levels of DNA methylation. The increase in DNA hypomethylation might arise from a possible role of G9a in re-initiating DNA methylation at hypomethylated sites after 5-Aza-CdR treatment. G9a might direct DNA methylation at such hypomethylated sites through recruitment of *de novo *DNMT3A/3B enzymes, similar to its role in ES cells [23]. Inhibition of G9a protein observed during 5-Aza-CdR treatment further supports a role of simultaneous inhibition of DNMTs and G9a in drug-induced DNA hypomethylation [48]. It is also possible that the increased DNA hypomethylation observed upon 5-Aza-CdR treatment of G9a knockdown cells might have arisen from an alteration in the cell growth rate leading to differential drug incorporation in DNA. 5-Aza-CdR has been previously shown to result in severe hypomethylation of rapidly dividing cells [49]. However, 5-Aza-CdR treated G9a-kd cells displayed a reduced growth rate compared to treated G9a WT cells ruling out such a possibility.

## Conclusions

Taken together, our data suggest that G9a is not required for preferential targeting of DNMT3A/3B to silent methylated domains and faithful maintenance of DNA methylation in differentiated somatic cells. However, G9a might serve as a potential target for combinatorial cancer treatment strategies involving DNMTs inhibitors to achieve greater drug-induced DNA hypomethylation and anti-proliferation effects.

## Methods

### Cell culture

HCT116 and 293T cells were maintained in McCoy's 5A and DMEM, respectively, containing 10% inactivated fetal bovine serum, 100 units/ml penicillin, and 100 μg/ml streptomycin. Puromycin was included in the culture medium at 3 μg/ml to maintain infected cells. 293T cells expressing different ΔDNMT3B isoforms were prepared as described previously [10]. For 5-Aza-CdR treatment experiments, cells were cultured in the presence of 0.3 μM 5-Aza-CdR for 24 h. After 24 h, the drug containing media was replaced with fresh media and cells were kept in culture till the mentioned time points. Cell counts were made using Coulter Counter (Beckman Coulter, Brea, CA).

### Nuclei preparation

Nuclei were prepared according to the procedure described previously [50]. Briefly, cells were trypsinized and washed once with PBS. The cells were then resuspended in ice-cold RSB buffer (10 mM Tris-HCl, pH 7.4, 10 mM NaCl, 3 mM MgCl_2_) containing protease inhibitors and kept on ice for 10 min before Dounce homogenization in the presence of 0.5% to 1% NP-40 to break up cell membranes. Nuclei were washed twice with RSB plus the protease inhibitors (Roche) without the detergent. Nuclear extracts were prepared by resuspending nuclei in RIPA buffer (50 mM Tris-HCl, pH 8.0, 150 mM NaCl, 1% NP-40, 0.5% DOC, 0.1% SDS) followed by sonication.

### MNase digestion and sucrose density gradient centrifugation

Sucrose density gradient experiments were performed as described previously [10]. Purified nuclei (1x10^8^) resuspended in 1 ml of RSB containing 0.25 M sucrose, 3 mM CaCl_2_, and 100 μM PMSF, were digested with 36 units of MNase (Worthington) for partial and 500 units of MNase (Worthington) for extensive digestion for 15 min at 37°C, and then the reaction was stopped with EDTA/EGTA (up to 10 mM). After microcentrifugation at 5,000 rpm for 5 min, the nuclear pellet was resuspended in 0.65 ml of the elution buffer (10 mM Tris-HCl, pH 7.4, 10 mM NaCl) containing 5 mM EDTA/EGTA, gently rocked for 1 hr at 4°C followed by microcentrifugation to obtain soluble nucleosomes. A quantity of 0.55 ml of soluble nucleosome containing buffer was fractionated through a sucrose density gradient solution (5% to 25% sucrose, 10 mM Tris-HCl, pH 7.4, 0.25 mM EDTA) containing NaCl of indicated concentrations, by centrifuging at 30,000 rpm for 16 h at 4°C. Fractions were taken from the top of the centrifuge tube to 16 aliquots. Proteins from same volume of each fraction (200 to 250 μl) were concentrated by TCA precipitation and subjected to western blot analysis. The EtBr treatment of the mononucleosome samples was done by adding 20 mg/ml EtBr to the samples (300 μg/ml at final), followed by incubation at room temperature for 10 min before loading onto the gradient.

### Lentiviral knockdown

The lentivirus particles containing N/S, shG9a5 and shG9a7 shRNA sequences were prepared using standard protocols. For lentivirus production, the vesicular stomatitis virus envelope protein G expression construct pMD.G1, the packaging vector pCMV ΔR8.91 and the transfer vector pHRCMVpuroSin8 were used as described previously [51]. Short hairpin RNA sequences encoding non-specific and sequences targeting *G9a *were as follows: N/S 5'- AAAACTGCAGAAAAAGGGTAGGTTCGACTAGCAGGACTCTTCTCTTGAA

AGAGTCTTGCTAGTTGAACCTACCCGGTGTTTCGTCCTTTCCACAAG-3'; shG9a5 5'- AAAACTGCAGAAAAAGACAGCAAGTCTGAAGTTGAAGCTCTCTCT

TGAAGAGCTTTAACTTCATACTTGCTGTCGGTGTTTCGTCCTTTCCACAAG -3' and shG9a7 5'-AAAACTGCAGAAAAAGGATGAATCTGAGAATCTTGAGGG

ATCTCTTGAATCCCTCCAGATTCTTAGATTCATCCGGTGTTTCGTCCTTTCCA

CAAG -3'. Infected HCT116 cells were selected in the presence of 3 μg/ml puromycin for two weeks.

### Western blot analysis

Proteins from the same volume of each fraction (200 to 250 μl) were concentrated by TCA precipitation, dissolved in SDS/β-mercaptoethanol loading buffer, and resolved on a 4% to 15% gradient SDS/PAGE gel (Bio-Rad, Hercules, CA). Antibodies against H3 (ab1791), DNMT3A (ab2850), and SUV39h1 (ab12405) were purchased from Abcam Inc. (Cambridge, UK); DNMT1 (sc-20701) and DNMT3B (sc-10235) from Santa Cruz Biotech (Santa Cruz, CA); Myc epitope tag (05-724) from Upstate (now Millipore, Billerica, MA) and G9a (G 6919); β-Actin (A 5316) from Sigma (Saint Louis, MO). The image of individual proteins was visualized using ECL detection system (Thermo Scientific, Waltham, MA and Millipore, Billerica, MA).

### DNA methylation analysis

Ms-SNuPE assay was performed as described previously [52,53]. Genomic DNA was prepared from HCT116 cells infected with either N/S, shG9a5, or shG9a7 constructs 14 days after lentiviral infection. To analyze the methylation status of individual DNA molecules, we cloned bisulfite PCR fragments of the loci of interest into the pCR2.1 vector using the TOPO-TA cloning kit (Invitrogen, Carlsbad, CA). Primer sequences are available on request. Individual colonies were screened for the insert and the region of interest was sequenced using M13 primers. DNA methylation levels were estimated by calculating the percentage of CpGs remaining methylated from the total number of CpGs assayed among all individual cells.

## Competing interests

The authors declare that they have no competing interests.

## Authors' contributions

SS, PAJ, and GL have made substantial contributions to conception and design. SS and HFH have made substantial contributions to acquisition of data. SS, GL, and PAJ have made contributions to analysis and interpretation of data. SS, DSG, HFH, SH, MRS, PAJ, and GL have been involved in drafting the manuscript or revising it critically for important intellectual content. SS, DSG, HFH, SH, MRS, PAJ, and GL have given final approval of the version to be published.

## Authors' information

SS, Ph.D; DSG, graduate student; HFH, graduate student, SJ, Ph.D (Research Assistant Professor); MRS, Ph.D (Professor and Department Chairman); PAJ, Ph.D (Professor and Director of Norris Comprehensive Cancer Center); GL, Ph.D and MD (Associate Professor of Research).

## Supplementary Material

Additional file 1 **Figure S1**. G9a and SUV39h1 strongly associate with mononucleosomes in both HCT116 and 293T cells. Mononucleosomal digests prepared by extensive MNase digestion of nuclei from **(A) **HCT116 cells and **(B) **293T cells were resolved by ultracentrifugation on a sucrose density gradient (5% to 25%) containing 300 mM NaCl. Gradients were fractionated and analyzed as described previously. The control lanes (denoted as C) on the gels were loaded with unfractionated nuclear extract loaded on the gels to monitor the quality of the immunostaining of the membranes. Click here for file

Additional file 2 **Figure S2**. Increased DNA hypomethylation of *MAGE-A1 *promoter in G9a knockdown cells upon treatment with 5-Aza-CdR. Methylation of *MAGE-A1 *promoter in G9a knockdown (shG9a5) and control (NS) HCT116 cells, treated with 5-Aza-CdR and PBS, was analyzed 72 h after drug treatment using bisulfite sequencing. CpG sites in the map of *MAGE-A1 *promoter are represented by the lower tick marks (top). Each straight line, with circles representing CpG sites, represents *MAGE-A1 *promoter sequence from a single cell (bottom). White circles indicate unmethylated CpG sites and black circles indicate methylated CpG sites. Cross indicates methylation status could not be determined. Residual DNA methylation levels were estimated by calculating the percentage of CpGs remaining methylated after drug treatment from the total number of CpGs assayed for the *MAGE-A1 *loci among all individual cells. Click here for file
