# Supplementary figures and images for: Lysine methyltransferase G9a is not required for DNMT3A/3B anchoring to methylated nucleosomes and maintenance of DNA methylation in somatic cells
Source: Epigenetics Chromatin. 2012 Jan 27;5:3. doi: 10.1186/1756-8935-5-3 (PMC3292817; doi:10.1186/1756-8935-5-3)

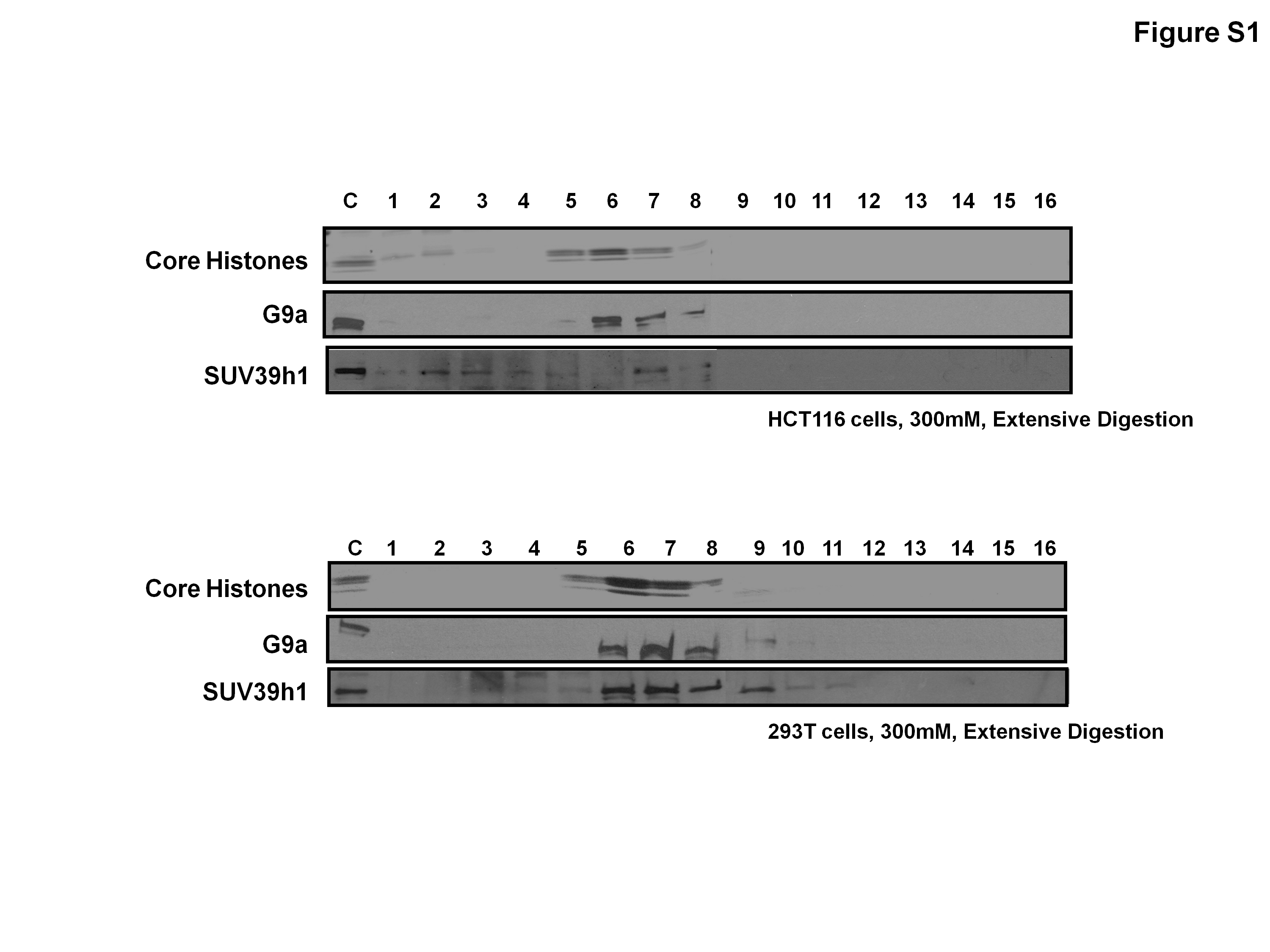

Supplement: Additional file 1 — Figure S1. G9a and SUV39h1 strongly associate with mononucleosomes in both HCT116 and 293T cells. Mononucleosomal digests prepared by extensive MNase digestion of nuclei from (A) HCT116 cells and (B) 293T cells were resolved by ultracentrifugation on a sucrose density gradient (5% to 25%) containing 300 mM NaCl. Gradients were fractionated and analyzed as described previously. The control lanes (denoted as C) on the gels were loaded with unfractionated nuclear extract loaded on the gels to monitor the quality of the immunostaining of the membranes. [file 1756-8935-5-3-S1.TIFF]

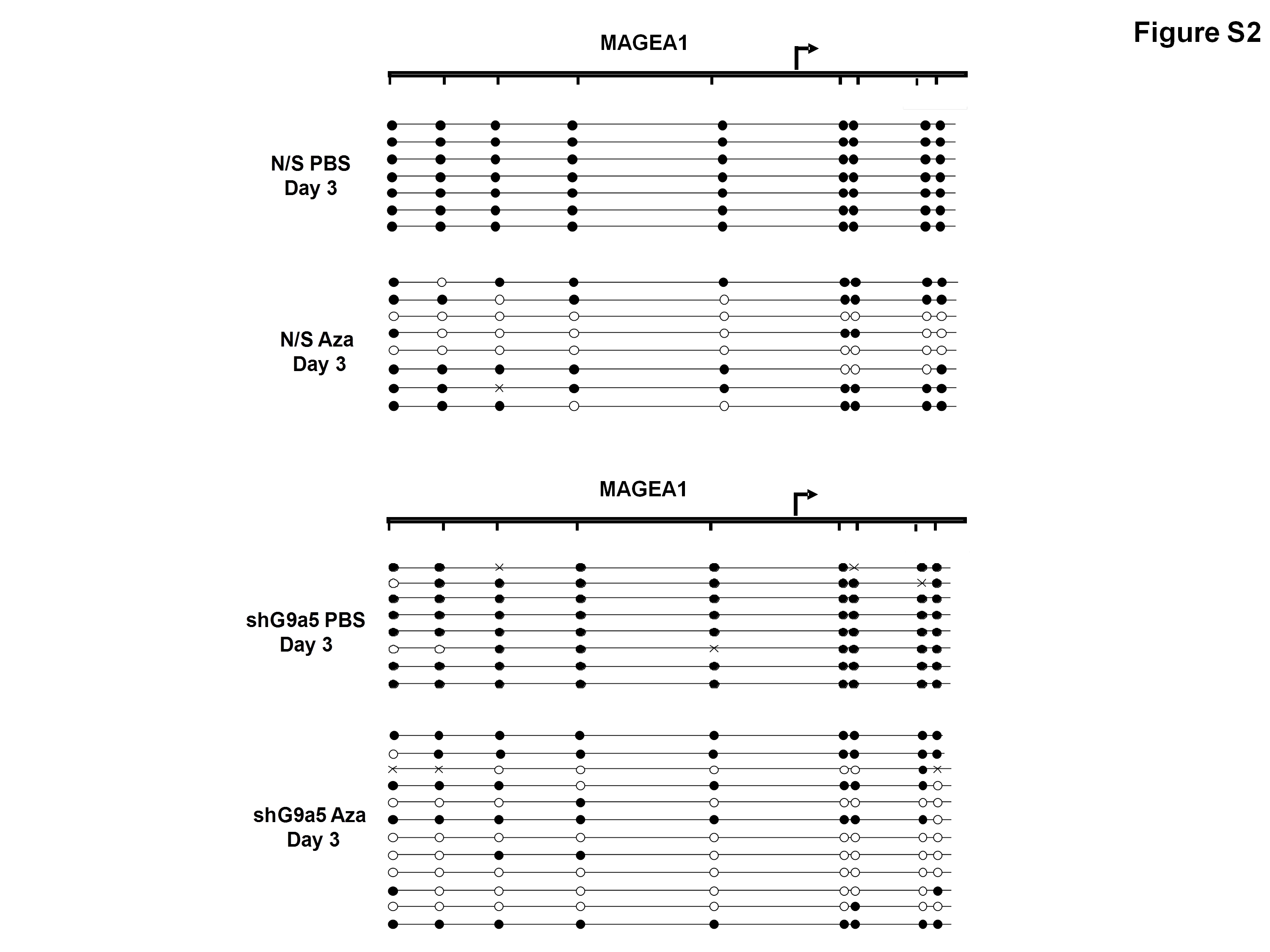

Supplement: Additional file 2 — Figure S2. Increased DNA hypomethylation of MAGE-A1 promoter in G9a knockdown cells upon treatment with 5-Aza-CdR. Methylation of MAGE-A1 promoter in G9a knockdown (shG9a5) and control (NS) HCT116 cells, treated with 5-Aza-CdR and PBS, was analyzed 72 h after drug treatment using bisulfite sequencing. CpG sites in the map of MAGE-A1 promoter are represented by the lower tick marks (top). Each straight line, with circles representing CpG sites, represents MAGE-A1 promoter sequence from a single cell (bottom). White circles indicate unmethylated CpG sites and black circles indicate methylated CpG sites. Cross indicates methylation status could not be determined. Residual DNA methylation levels were estimated by calculating the percentage of CpGs remaining methylated after drug treatment from the total number of CpGs assayed for the MAGE-A1 loci among all individual cells. [file 1756-8935-5-3-S2.TIFF]
